# NanoString Digital Molecular Profiling of Protein and microRNA in Rhabdomyosarcoma

**DOI:** 10.3390/cancers14030522

**Published:** 2022-01-21

**Authors:** Atif A. Ahmed, Midhat S. Farooqi, Sultan S. Habeebu, Elizabeth Gonzalez, Terrie G. Flatt, Ashley L. Wilson, Frederic G. Barr

**Affiliations:** 1 Department of Pathology and Laboratory Medicine, Children’s Mercy Hospital, Kansas City, MO 64108, USA; 2 Department of Pathology and Laboratory Medicine, Children’s Mercy Hospital/University of Missouri, Kansas City, MO 64108, USA; msfarooqi@cmh.edu (M.S.F.); smhabeebu@cmh.edu (S.S.H.); 3 Department of Pediatric Hematology-Oncology, Children’s Mercy Hospital/University of Missouri, Kansas City, MO 64108, USA; egonzalezdominguez@cmh.edu (E.G.); tgflatt@cmh.edu (T.G.F.); 4 Seattle Children’s Research Institute, Seattle, WA 98101, USA; ashley.wilson2@seattlechildrens.org; 5 Laboratory of Pathology, National Cancer Institute, Bethesda, MD 20892, USA; frederic.barr@nih.gov

**Keywords:** rhabdomyosarcoma, PI3K/AKT, NanoString, digital spatial profiling, microRNA

## Abstract

**Simple Summary:**

NanoString digital profiling methods are novel techniques to identify biologic markers from human formalin-fixed, paraffin-embedded cancer tissue. We have applied NanoString Digital spatial profiling and microRNA profiling methods in non-alveolar rhabdomyosarcoma, a common soft tissue tumor in young adults and children with variable prognosis. Our results have highlighted aberrant miRNA expression and over-expression of several members of PI3-AKT, MAPK and apoptosis signaling pathways in fusion-negative rhabdomyosarcoma, particularly in tumors with unfavorable prognosis. INPP4B, an entry molecule in the PI3/AKT pathway, was significantly over-expressed in tumors with poor prognosis, confirmed by traditional immunohistochemistry. Several microRNAs had increased expression in association with poor patient prognosis. These results highlight the utility of NanoString digital profiling as a screening method to identify prognostic biomarkers of interest in rhabdomyosarcoma from formalin-fixed paraffin-embedded tissue.

**Abstract:**

Purpose: Rhabdomyosarcoma (RMS) exhibits a complex prognostic algorithm based on histologic, biologic and clinical parameters. The embryonal (ERMS) and spindle cell-sclerosing RMS (SRMS) histologic subtypes warrant further studies due to their heterogenous genetic background and variable clinical behavior. NanoString digital profiling methods have been previously highlighted as robust novel methods to detect protein and microRNA expression in several cancers but not in RMS. Methods/Patients: To identify prognostic biomarkers, we categorized 12 ERMS and SRMS tumor cases into adverse (*n* = 5) or favorable (*n* = 7) prognosis groups and analyzed their signaling pathways and microRNA profiles. The digital spatial profiling of protein and microRNA analysis was performed on formalin-fixed, paraffin-embedded (FFPE) tumor tissue using NanoString technology. Results: The detectable expression of several component members of the PI3K/AKT, MAPK and apoptosis signaling pathways was highlighted in RMS, including INPP4B, Pan-AKT, MET, Pan-RAS, EGFR, phospho-p90 RSK, p44/42 ERK1/2, BAD, BCL-XL, cleaved caspase-9, NF1, PARP and p53. Compared to cases with favorable prognosis, the adverse-prognosis tumor samples had significantly increased expression of INPP4B, which was confirmed with traditional immunohistochemistry. The analysis of microRNA profiles revealed that, out of 798 microRNAs assessed, 228 were overexpressed and 134 downregulated in the adverse prognosis group. Significant over-expression of oncogenic/tumor suppressor miR-3144-3p, miR-612, miR-302d-3p, miR-421, miR-548ar-5p and miR-548y (*p* < 0.05) was noted in the adverse prognosis group. Conclusion: This study highlights the utility of NanoString digital profiling methods in RMS, where it can detect distinct molecular signatures with the expression of signaling pathways and microRNAs from FFPE tumor tissue that may help identify prognostic biomarkers of interest. The overexpression of INPP4B and miR-3144-3p, miR-612, miR-302d-3p, miR-421, miR-548y and miR-548ar-5p may be associated with worse overall survival in ERMS and SRMS.

## 1. Introduction

Rhabdomyosarcoma (RMS), the most common pediatric soft tissue sarcoma, is clinically, genetically and histologically a heterogeneous tumor that has been classified into three major histologic types: embryonal (ERMS), alveolar and spindle cell-sclerosing (SRMS). While alveolar RMS is a more aggressive disease depending on the presence of *PAX3* or *PAX7* with *FOXO1* gene fusions, the ERMS and the SRMS types are biologically heterogeneous and exhibit variable clinical behavior and a wide spectrum of genetic alterations, suggesting the presence of additional biologic prognostic modulators [1,2,3,4]. Genetic studies have disclosed mutations and subsequent activation of the phosphoinositide 3-kinase/AKT (PI3K/AKT) and the mitogen activated protein kinase (MAPK) signaling pathways in RMS that contribute to tumor growth, cellular proliferation and invasion and may affect patients’ prognosis and outcome [5]. In particular, mutations in the *RAS* family members, *FGFR4*, *PIK3CA* and *CTNNB1* have been identified in ERMS. PI3K/AKT signaling pathway mutations were also detected in a clinically aggressive subset of SRMS in association with *MYOD1* gene mutations [6]. However, protein expression profiles of individual pathway components in ERMS and SRMS have not been clearly and thoroughly delineated. The stimulation of signaling pathways is linked to the loss of myogenic differentiation and altered expression of microRNA (miRNA) molecules [7]. These small non-coding RNAs play important roles in RMS pathogenesis through regulation of myogenic differentiation. Both myogenic and non-muscle specific miRNAs have been implicated in RMS development and are postulated to influence tumor behavior and patient outcome [8].

Digital profiling, offered by NanoString Technologies (Seattle, WA, USA: www.nanostring.com, accessed on 24 September 2021), represents a set of molecular techniques and robust analytic tools that characterize of the functional genomic and proteomic landscape of cancer from all types of specimens. Digital spatial profiling (DSP) has the advantage that it can be performed on formalin-fixed, paraffin-embedded (FFPE) tissue sections, thus maximizing the benefit from scant cancer tissue [9]. Few studies have highlighted DSP role in studying the immune environment proteins and cells, particularly in the context of immunotherapy targets against cancer [10]. Another highly multiplexed technique, NanoString nCounter microRNA assay allows for direct digital detection and quantification of hundreds of human miRNAs and has been validated for RNA extracted from FFPE tissue [11]. Both techniques are based on digital read-out of hybridized oligonucleotide probes [9]. In the few cancers that have been studied, DSP and miRNA analysis offer good sensitivity, reproducibility and utility for clinical applications [12,13,14]. In this study, we have analyzed a set of ERMS and SRMS for their miRNA and signaling pathway protein expression profiles as measured by NanoString digital profiling methods. Our study reveals high expression of several PI3K/AKT, MAPK and apoptosis pathway molecules in RMS, with significant differences in expression levels of some of these proteins in tumors with poor prognosis. We have also used traditional immunohistochemistry to confirm expression of proteins outlined by DSP. Few miRNAs also exhibit differential expression in the poor prognosis group.

## 2. Materials and Methods

A retrospective analysis of archived paraffin-embedded tissue was approved by the Institutional Research Integrity Board of Children’s Mercy Hospital, Kansas City.

### 2.1. Patients/Subjects/Samples

**Figure 1:**
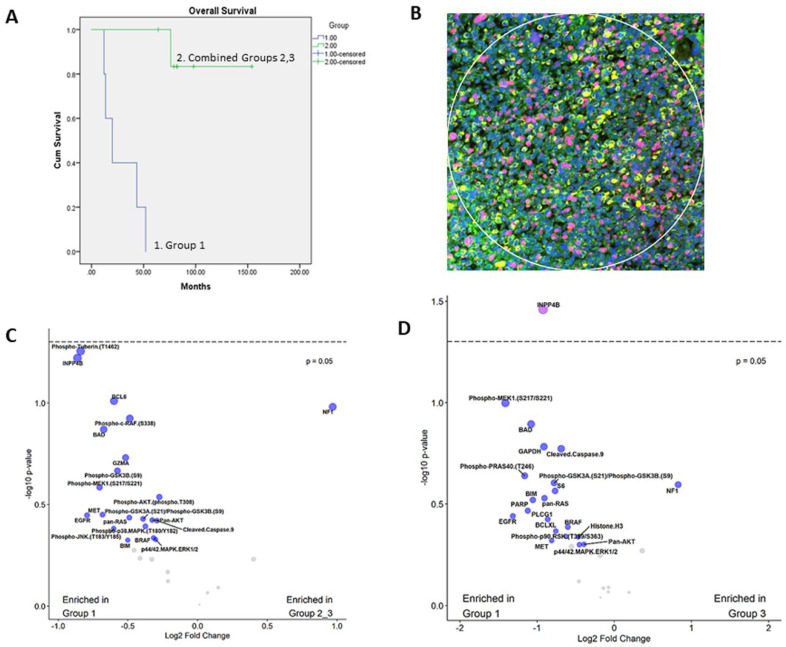
Differences between Group 1 and Group 2 patients in overall survival and expression of pathway members: (**A**) Kaplan–Meier survival plot reveals significant difference in overall survival between Group 1 and Group 2, 3 (combined) patients. (**B**) A representative image of a region of interest (ROI). ROIs were selected based on immunofluorescence staining with desmin (yellow), Ki-67 (red), cytokeratin (green-absent) and DNA (blue). (**C**) A volcano plot showing differential expression of analytes and comparison between Group 1 and Groups 2 and 3 tumors (combined), as measured by DSP. (**D**) A volcano plot showing differential expression of analytes and comparison between Group 1 and Group 3 tumors, as measured by DSP. Dotted line represents cut-off *p*-value of 0.05. Gray dots represent remaining analytes with no significant differences.

Tumor cases were collected from the Pathology archives over a period of 20 years (1999–2019). Patients up to 18 years of age at the time of the surgery with the pathologic diagnosis of ERMS or SRMS were included. The main inclusion criterion included the availability of FFPE blocks that contain more than 90% tumor. Sections of tumor mixed with normal tissue were excluded. Diagnoses were confirmed by re-review of the histologic slides for the characteristic morphology and immunophenotype (Table 1). Patients and tumors were selected from our previous study [15] and were classified into three prognostic and histologic groups, based on clinical follow-up information and calculation of overall survival (Figure 1A). Group 1 was defined as tumors with adverse or poor prognosis and contained both ERMS and SRMS. Group 2 tumors were favorable prognosis SRMS while Group 3 tumors were favorable prognosis ERMS.

### 2.2. NanoString Digital Spatial Profiling

Formalin-fixed paraffin-embedded (FFPE) sections from 12 RMS tumor cases were subjected to highly multiplexed DSP of proteins using NanoString GeoMx^®^ Digital Spatial Profiler (https://www.nanostring.com/products/geomx-digital-spatial-profiler/geomx-dsp-overview, accessed on 24 September 2021) according to previously described methods [12,14]. Briefly, indexing oligonucleotides were covalently attached to primary antibodies with a UV-photocleavable (PC) linker. The profiling process began on a slide-mounted FFPE tissue section that underwent antigen retrieval and incubation with a cocktail of PC-oligo-labeled primary antibodies. The same tissue section was also stained with four fluorescently labeled imaging reagents (desmin, pan-cytokeratin, Ki-67 and DNA) to identify tissue features and tumor regions of interest (ROI) versus stromal cells (Figure 1B). After incubation and hybridization, slides were loaded into the DSP instrument and each sample was scanned to produce a digital image of the tissue morphology based on the fluorescent markers. Scanned images were used to guide the selection of ROIs for profiling the PC-oligo-conjugated antibodies that were subsequently illuminated and released by UV light. The released indexing oligonucleotides were collected and digitally counted.

Targets of interest measured by this assay included a previously validated panel consisting of signaling components of the PI3K/AKT, MAPK and apoptosis signaling pathways. ROI in the tumor sections were selected based on high Ki-67 proliferative areas, positive staining for desmin and negative staining with pan-cytokeratin. The latter markers are clinically used to confirm or exclude RMS diagnosis, respectively. Digital raw counts were measured with the nCounter digital analyzer and analyzed with GeoMx DSP data center. Normalization of raw data was achieved with housekeeping proteins and immunoglobulin IgG as controls. Background correction and measurement of signal intensity in relation to background noise was performed for each protein. Read-out of protein expression was estimated using signal to noise ratio (SNR) and quantified as 1–3 (SNR = 1 indicates protein signal similar to background noise; SNR = 3 indicates signal is three times the background noise).

### 2.3. NanoString MicroRNA Profiling

Nucleic acid was retrieved from FFPE RMS tumor tissue scrolls. Total RNA was extracted using the Qiagen RNeasy FFPE kit. RNA concentrations ranged from 266.4–1680.8 ng/uL. RNA with a 260/230 nm absorbance ratio of >1.8 and 260/280 nm absorbance ratio >1.8 was used for subsequent experiments on the NanoString nCounter platform according to previously described method [13]. Briefly, sequence-specific oligonucleotide probes tagged with fluorescent barcodes were used to bind to and digitally measure small RNAs. The abundance of specific capture probe-bound mature miRNA molecules was measured using the nCounter digital analyzer to count individual fluorescent barcodes. The NanoString miRNA pre-designed panel simultaneously detects >800 human miRNAs, including five housekeeping transcripts. Positive and negative proprietary spike-in controls, hybridization controls and ligation-specific controls were included to determine sample integrity, quality and background. All data analysis was performed using nSolver and ROSALIND software. Stringent normalization of miRNA data was achieved by eliminating digital counts below 50 and counts with high variability (%CV > 90). Comparison of miRNA profiles between different groups was performed, and heat maps and ratio tables with statistically significant differences were generated.

### 2.4. Immunohistochemistry

Automated immunohistochemistry for INPP4B was performed on each case with a Leica Bond instrument. A monoclonal antibody (ab81269 from Abcam, Cambridge, MA, USA) that detects the N-terminal of human INPP4B was used in 1:50 dilution. Experiments were performed with antigen retrieval and visualization with a brown dye, according to manufacturer specifications. Breast and colon cancer tissue served as positive controls. Percentage of tumor cells with positive staining was compared between the prognostic groups. Negative controls were similarly treated except for omission of primary 2.5. Statistical Analysis

Kaplan–Meier survival curves were plotted to estimate the difference in overall survival between the prognostic tumor groups. Student’s t-test was calculated to estimate the statistical significance of the differences in the INPP4B immunohistochemistry scores of each group, assuming a normal distribution. Student’s t-test was also utilized as part of the NanoString analysis software.

## 3. Results

### 3.1. Prognostic Classification of Patients and Tumor Specimens

A total of 12 RMS cases were selected, including 5 ERMS and 7 SRMS. ERMS tumors showed the cellular proliferation of ovoid or round cells with hyperchromatic nuclei and abundant mitosis in myxoid or scant stroma. SRMS tumors were characterized by spindle cell proliferation arranged in whorls and fascicles in variably sclerotic stroma. Two ERMS tumors had evidence of anaplasia with large cells and bizarre-looking nuclei. All tumors exhibited immunoreactivity with myogenin and desmin and received standard RMS treatment. Patient demographics and clinical features are summarized in Table 1. Five tumors exhibited poor prognosis (Group 1) and seven had favorable prognosis (Groups 2 and 3). Kaplan–Meier analysis revealed patients in Group 1 had an adverse prognosis with a statistically significant difference in the overall survival, *p* < 0.0001 (Figure 1A).

### 3.2. Expression of PI3K-AKT, MAPK and Apoptosis Pathways

Several proteins were identified that had a signal to noise ratio (SNR) of more than three indicating high expression, including INPP4B, Pan-AKT, MET, Pan-RAS, EGFR, phospho-p90 RSK, p44/42 ERK1/2, BAD, BCL-XL, cleaved caspase-9, NF1, PARP and p53 (Table 2). Compared to the other groups, Group 1 tumors were enriched for proteins in the PI3K/AKT, MAPK and apoptosis pathways. Interestingly, INPP4B, an early member of the PI3K/AKT proliferation pathway, had higher expression in Group 1 tumors compared to Groups 2 and 3 (Figure 1C). Compared to group 3, INPP4B (*p* = 0.0363) and phospho-MEK1 (*p* = 0.1) were notably overexpressed in Group 1 (Figure 1D). Further analysis of INPP4B revealed the presence of a rare case in group 2 (case 7, Table S1) with relatively high expression that is incongruent with other group members. No other statistically significant differences were identified in the expression of the remaining markers (Figure S1A–C).

### 3.3. Immunohistochemical Expression of INPP4B

**Figure 2:**
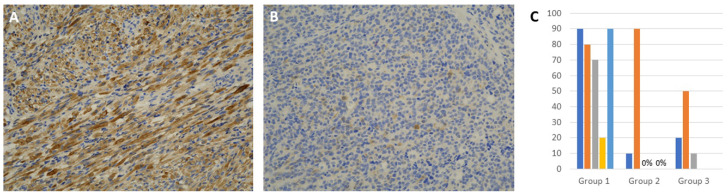
Immunohistochemistry for INPP4B: (**A**) Representative image of an SRMS from Group 1 showing strong, diffuse cytoplasmic immunoreactivity (×200). (**B**) Representative image from a Group 3 ERMS where tumor cells were mostly negative, except for a few scattered positive cells (×200). (**C**) A chart showing percentage immunoreactivity of INPP4B in individual samples.

Positive cytoplasmic staining with INPP4B was noted with variable percentage of stained cells. The staining was more prevalent in Group 1 than Groups 2 and 3 tumors (Figure 2). All tumors in Group 1 had higher staining (5/5) that ranged from 20–90% of tumor cells (mean 70%) compared to tumors in the other groups that showed staining in 5 out of 7 tumors (range 0–90%, mean 25.71%), and the difference was statistically significant at *p* = 0.0376 (Table 1). There was no significant difference between Groups 2 and 3 tumors. A detailed analysis revealed the presence of outliers, such as case 4 in Group 1 and case 2 in Group 2 (Figure 2C).

### 3.4. MiRNA Profiling Showing Differential Expression in RMS Groups

**Figure 3:**
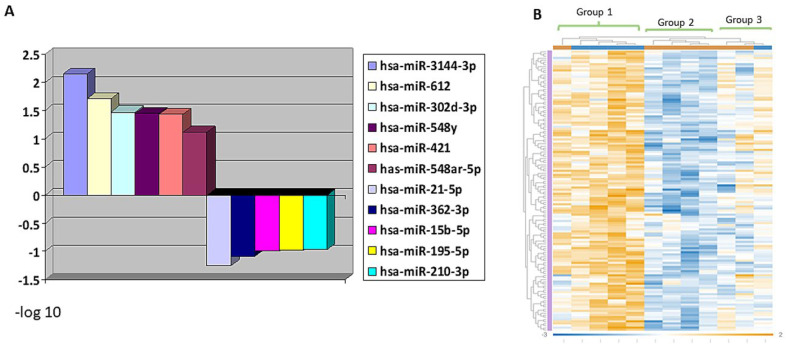
Differential expression of miRNA. (**A**) The topmost significantly altered miRNA expression according to p value (Y axis = −log 10). (**B**) Heat map revealing differential expression and clustering of miRNAs expression in each RMS group.

Differential expression of miRNAs was observed in Group 1 versus Groups 2 and 3. Out of 798 miRNAs assessed, 228 had increased and 134 had decreased expressions in the adverse prognosis group. miR-3144-3p, miR-612, miR-302d-3p, miR-548y, miR-421, miR-548y and miR-548ar-5p were significantly overexpressed in Group 1 tumors compared to Group 2 and 3 (combined) (*p* < 0.05) (Figure 3). In addition, SRMS (Group 2) showed the differential downregulation of 208 and over-expression of 100 miRNAs in comparison to ERMS (Group 3). miR-26b-5p and miR-331-3p were significantly downregulated in SRMS (*p* < 0.05), while miR-15a-5p was overexpressed (*p* = 0.0881), compared to Group 3 ERMS. Heat map graphic representation of the top 134 differentially expressed genes is shown in Figure 3. The top 30 genes predicted to affect miRNA expression (Table S2) are mostly related to the regulation of transcription and methylation.

## 4. Discussion

NanoString’s digital profiling methods have detected expression of the PI3K/AKT, MAPK and apoptosis pathways and differential expression of miRNAs in RMS subsets, suggesting they may have a possible role in influencing RMS biologic behavior. The results obtained from FFPE tumor profiling are similar to what has been reported via phosphoprotein pathway mapping and integrated genomic-proteomic analysis [16,17]. The differential miRNA expression and pathway activation of PI3K/AKT and MAPK have been previously linked to invasion and metastasis and are often targeted for experimental therapy [16,17]. Through extensive crosstalk and feedback, PI3K/AKT and MAPK signaling pathways regulate each other to activate proliferation and protect tumor cells from apoptosis [18,19,20]. An earlier study has revealed pathway activation in RMS, regardless of the histologic type [5]. Phosphorylated Akt Ser^473^, 4EBP1 Thr^37/46^, eIF4G Ser^1108^ and p70S6 Thr^389^ were significantly upregulated in a set of poor prognosis RMS [5]. In contrast, our profiling experiments, limited to ERMS and SRMS, revealed the expression of several pathway components, including INPP4B, which was significantly overexpressed in tumors with poor prognosis. INPP4B is an early entry molecule to the PI3K/AKT pathway and hence positions itself as an ideal prognostic marker in RMS. It acts as a phosphoinositide phosphatase that is required for the engagement and recruitment of PI3K through the activation of AKT or SGK3. INPP4B may have a dual oncogenic/tumor suppressor role and was found as an oncogenic regulator in colon cancer, acute myeloid leukemia and in a subset of melanomas [21,22]. The confirmation of higher INPP4B expression in Group 1 tumors by traditional immunohistochemistry adds to the validity of DSP methods as a useful screening tool for biomarkers of interest in RMS [23]. Nevertheless, DSP has the advantage that it can circumvent the problems of subjective interpretation and difficult standardization frequently encountered during routine immunohistochemical antibody optimization. DSP allows multiplex testing and correlation with morphology through ROI selection, thus providing more valuable and accurate information than blind biologic experimentation on frozen/fresh tumor samples.

The DSP experiments have confirmed detectable expression of other signaling proteins, albeit with no significant differences between the prognostic groups (Table 1). EGFR and MET are receptor proteins that activate PI3K/AKT and MAPK signaling pathways downstream and are known to play major roles in RMS progression. Both proteins are considered as experimental targets of inhibitory therapy [24,25,26]. Furthermore, over-expression of RAS is linked to RAS mutations, which have been identified as the most common oncogenic genetic aberration in fusion-negative RMS [27]. Our study also found that phospho-p90 RSK and p44/42 ERK1/2 were differentially expressed between the prognosis groups. Future work is needed to further elucidate the roles of these proteins in RMS. 

Apoptosis is one of the most commonly studied pathways in RMS, as defects in apoptotic programs or the aberrant expression of apoptotic proteins may result in chemotherapy resistance [19]. Our DSP experiments revealed the high expression of several members of the apoptosis pathway, including proapoptotic (BAD), anti-apoptotic (BCL-XL), caspase 9 and tumor suppressor proteins that also promote apoptosis (NF1, PARP and p53). It has been found that the upregulation of BCL-XL inhibits apoptosis and leads to RMS sarcomagenesis by activation of MAPK signaling pathway [17,28]. On the other hand, apoptosis can be induced as a form of experimental treatment by specific miRNAs that suppress tumor growth [29,30,31].

MicroRNAs can have various physiologic and neoplastic effects, and their expression is regulated by transcriptional and post-transcriptional mechanisms, all of which can lead to activation of myogenic transcription factors and PI3K/AKT and MAPK signaling pathways [32]. In RMS, miRNAs can play crucial roles as tumor suppressor genes, as well as oncogenes. Their dysregulation leads to the modulation of molecular signaling pathways involved in RMS biology and can therefore affect tumor behavior and patient prognosis [32]. Our miRNA profiling experiments identified the significant overexpression of miR-3144-3p, miR-612, miR-302d-3p, miR-421 and miR-548 in the poor prognosis group, thus hinting to their potential role as prognostic indicators. Few preliminary studies have highlighted their oncogenic and/or tumor suppressor roles in alignment with their predicted gene functions [33,34,35,36]. Furthermore, miR-421 may have an additional role as a predictor of unfavorable prognosis in esophageal cancer [37]. Although the miRNA findings in this study were not confirmed by independent in vitro or functional studies, NanoString digital profiling allows for miRNA analysis from stored and archived tumor tissue that has already been microscopically examined, thus correlating morphology with biology. NanoString digital methods and nCounter analysis have been rigorously validated and commercially available for more than a decade, eliminating the need for additional confirmation [38]. 

## 5. Conclusions

In conclusion, this study highlights the utility of NanoString digital molecular profiling methods and their potential for screening and identifying relevant proteins and miRNAs from formalin-fixed paraffin-embedded pediatric cancer tissues. This study is limited by the small number of patients due to the difficulty in procuring adequate tissue samples that contain pure tumor. The small patient cohort decreases the statistical power of the findings and limits the study usefulness and full utilization. Cancer specimens from pediatric patients can be scant and are mostly utilized to establish the pathologic diagnosis limiting benefit from biologic studies. NanoString technology can avail the use of such scant tissue generating more valuable information. Unfavorable prognosis in a subset of RMS tumors may be linked to the differential expression of specific miRNAs and the over-expression of PI3K/AKT, MAPK and apoptosis pathway members, particularly INPP4B. However, due to the limited number of patients in this study, functional experiments with cell lines and/or additional studies with higher number of cases may be needed to confirm the prognostic role of INPP4B and miRNA in RMS. 

## Figures and Tables

**Table 1:** Clinicopathologic features of RMS cases selected for NanoString digital profiling.

	Group 1 (*n* = 5)	Group 2 (*n* = 4)	Group 3 (*n* = 3)
Age (years)	2–15	5–18	5–8
Male: female ratio	4:1	3:1	3:0
Tumor histology	3 SRMS, 2 ERMS	4 SRMS	3 ERMS
Overall survival (months)	Range: 5–52 Mean: 28.2	Range: 64–82 Mean: 141	Range: 82–154 Mean: 111
Mortality rate	5/5	1/7	0/3
INPP4B, IHC positive rate	Range: 20–90% Mean: 70	Range: 0–90% Mean: 29.15	Range: 0–50 Mean: 33.09

**Table 2:** Expression levels of signaling pathway proteins in RMS (all groups combined) as determined by DSP analysis of signal to noise ratio (SNR).

SNR Expression Levels	PI3K/AKT	MAPK	Apoptosis
SNR > 3	INPP4B	Pan-Ras	BAD
	Pan-AKT	EGFR	Cleaved caspase-9
	MET	phospho-p90 RSK	BCLXL
		p44/42 MAPK ERK1/2	NF1
			PARP
			P53
SNR > 1	phospho-PRAS40	BRAF	BIM
	phospho-GSK3A	Phospho-MEK1	
	Phospho-AKT1		
	PLCG1		
SNR < 1	Phospho-AKT	Phospho-cRAF	BCL-6
	Phospho-tuberin	Phospho-p44/42 MAPK ERK1/2	CD95/Fas
	phospho-GSK3B	Phospho-JNK	
		Phospho-p38 MAPK	

## Data Availability

Data are available from the authors upon reasonable request and will be anonymized to comply with HIPAA and IRB regulations.

## Supplementary Materials

The following supporting information can be downloaded at: https://www.mdpi.com/article/10.3390/cancers14030522/s1, Figure S1: Box data illustrating expression of pathway components in the tumor groups, as illustrated by NSR values. A. PI3K-AKT components in Group 1 versus combined Groups 2 and 3; B. MAPK components in Group 1 versus combined Groups 2 and 3; C. Cell death module in Group 1 versus combined Groups 2 and 3. Table S1: DSP normalized digital counts of INPP4B in the different cases and controls. High counts in smooth muscle actin (SMA) corresponds to its known immunohistochemical expression in rhabdomyosarcoma. Low counts in pancytokeratin (PanCk) corresponds to its absence of expression in rhabdomyosarcoma. Case 7 of Group 2 revealed high expression, similar to Group 1. Table S2: Predicted affected genes in association with the involved miRNA’s.
