# Supplementary material for: NanoString Digital Molecular Profiling of Protein and microRNA in Rhabdomyosarcoma
Source: Cancers (Basel). 2022 Jan 21;14(3):522. doi: 10.3390/cancers14030522 (PMC8833805; doi:10.3390/cancers14030522)
Supplement: Supplementary file 1 [file cancers-14-00522-s001.zip › Figure S1.pdf]

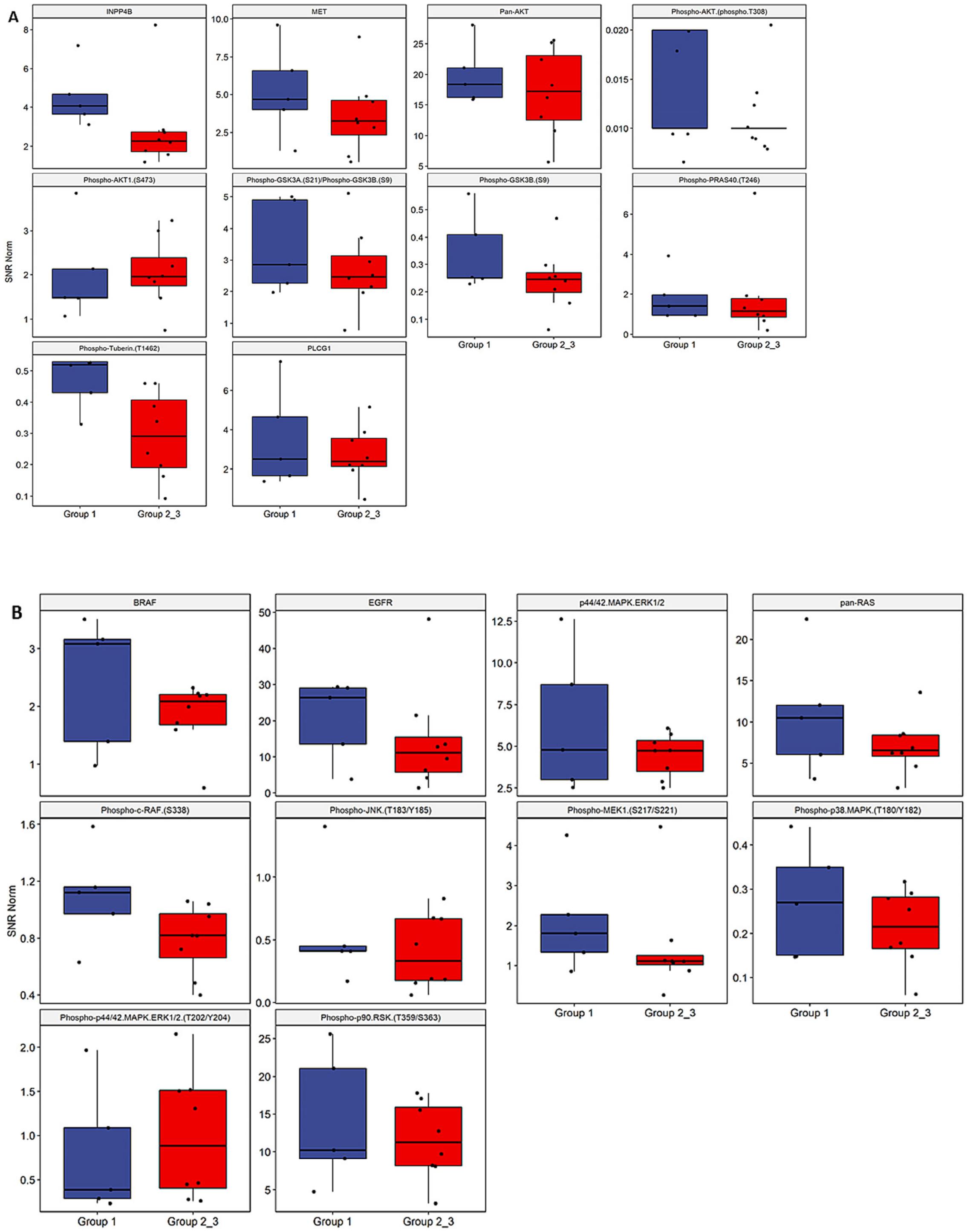
A
INPP4B
MET
Pan-AKT
Phospho-AKT.(phospho.T308)
Phospho-AKT1.(S473)
Phospho-GSK3A.(S21)/Phospho-GSK3B.(S9)
Phospho-GSK3B.(S9)
Phospho-PRAS40.(T246)
Phospho-Tuberin.(T1462)
PLCG1
SNR Norm
Group 1
Group 2_3
B
BRAF
EGFR
p44/42.MAPK.ERK1/2
pan-RAS
Phospho-c-RAF.(S338)
Phospho-JNK.(T183/Y185)
Phospho-MEK1.(S217/S221)
Phospho-p38.MAPK.(T180/Y182)
Phospho-p44/42.MAPK.ERK1/2.(T202/Y204)
Phospho-p90.RSK.(T359/S363)
SNR Norm
Group 1
Group 2_3

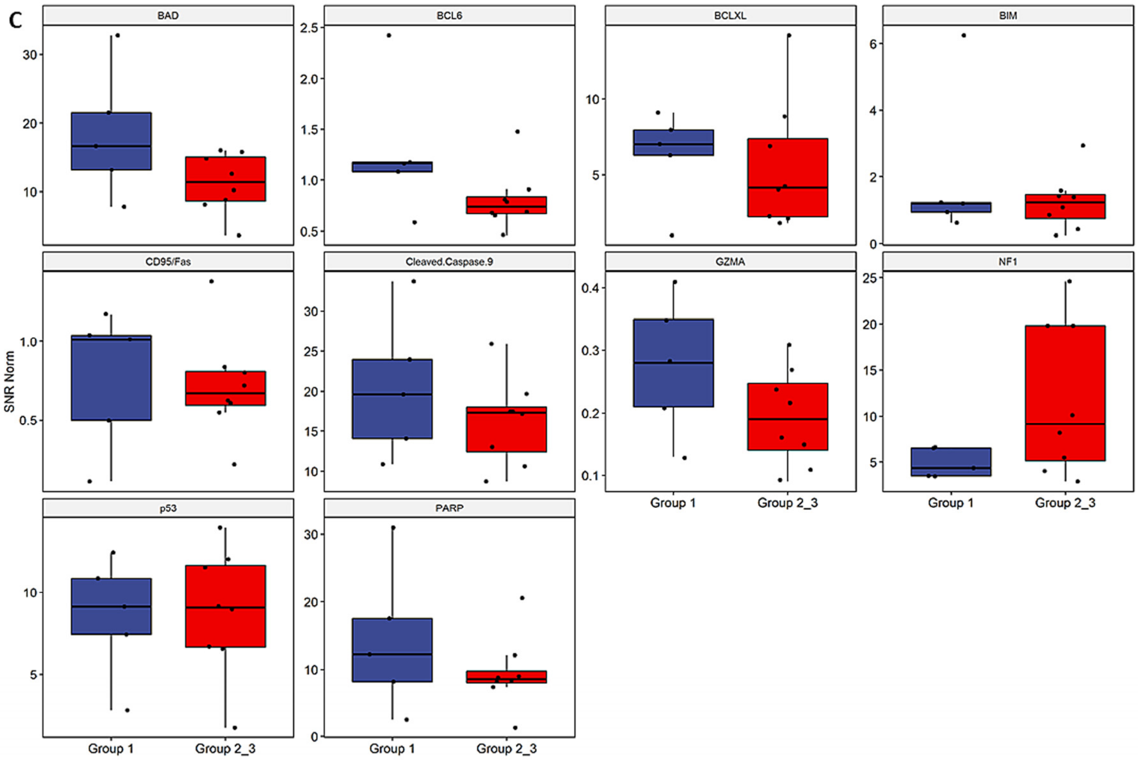


**Figure S1.** Box data illustrating expression of pathway components in the tumor groups, as illustrated by NSR values. A. PI3K-AKT components in Group 1 versus combined Groups 2 and 3; B. MAPK components in Group 1 versus combined Groups 2 and 3; C. Cell death module in Group 1 versus combined Groups 2 and 3.
