# Supplementary material for: NanoString Digital Molecular Profiling of Protein and microRNA in Rhabdomyosarcoma
Source: Cancers (Basel). 2022 Jan 21;14(3):522. doi: 10.3390/cancers14030522 (PMC8833805; doi:10.3390/cancers14030522)
Supplement: Supplementary file 1 [file cancers-14-00522-s001.zip › Table S1.pdf]

**Supplementary Table S1**

DSP normalized digital counts of INPP4B in the different cases and controls. High counts in smooth muscle actin (SMA) corresponds to its known immunohistochemical expression in rhabdomyosarcoma. Low counts in pancytokeratin (PanCk) corresponds to its absence of expression in rhabdomyosarcoma. Case 7 of Group 2 revealed high expression, similar to Group 1.

| Case ID | GAPDH | Histone H3 | Ms IgG1 | Ms IgG2a | INPP4B | PanCk | SMA |
|---|---|---|---|---|---|---|---|
| 1 | 314.36 | 344.68 | 0.69 | 0.64 | 7.19 | 0.87 | 195.94 |
| 2 | 66.95 | 537.19 | 0.52 | 0.59 | 2.33 | 0.74 | 121.15 |
| 3 | 56.69 | 124.42 | 0.38 | 0.48 | 1.57 | 0.54 | 569.8 |
| 4 | 256.62 | 553.75 | 0.75 | 0.74 | 4.67 | 0.88 | 41.95 |
| 5 | 117.97 | 654.62 | 0.69 | 0.71 | 2.71 | 0.83 | 25.74 |
| 6 | 294.78 | 639 | 0.67 | 0.66 | 4.06 | 0.91 | 31.55 |
| 7 | 429.92 | 402.66 | 0.76 | 0.58 | 8.24 | 0.81 | 141.89 |
| 8 | 217.07 | 456.71 | 0.64 | 0.64 | 2.82 | 0.93 | 224.03 |
| 9 | 184.64 | 329.9 | 0.79 | 0.48 | 3.64 | 1.09 | 129.19 |
| 10 | 149.77 | 647.64 | 0.58 | 0.6 | 1.76 | 0.69 | 28.45 |
| 11 | 99.6 | 518.23 | 0.82 | 0.69 | 3.1 | 0.86 | 7.91 |
| 12 | 166.08 | 393.05 | 0.67 | 0.6 | 1.19 | 0.74 | 227.18 |

Group 1: case ID 1, 4, 6, 9 , 11. Group 2: Case ID 2, 7, 10, 12. Group 3: Case ID 3, 5, 8
