# Supplementary material for: NanoString Digital Molecular Profiling of Protein and microRNA in Rhabdomyosarcoma
Source: Cancers (Basel). 2022 Jan 21;14(3):522. doi: 10.3390/cancers14030522 (PMC8833805; doi:10.3390/cancers14030522)
Supplement: Supplementary file 1 [file cancers-14-00522-s001.zip › Table S2.pdf]

Supplementary Table S2. Predicted affected genes in association with number of involved miRNA's.

Gene	Number of miRNA's
KMT2C	72
TNRC6B	70
NFAT5	66
ONECUT2	65
QKI	65
NFIB	65
INO80D	62
CELF2	60
LCOR	59
USP9X	58
NTRK3	58
NUFIP2	58
NOVA1	57
TNRC6A	56
UBN2	56
ZNF148	56
IKZF2	55
CBX5	55
NCR3LG1	54
CNOT6L	53
ATXN1	53
ZFHX4	53
TNPO1	53
REV3L	53
MECP2	52
BNC2	52

POU2F1	52
UBE2W	52
TANC2	52
PTEN	52
